# Medical Items

**Published:** 1916-02

**Authors:** 


### MEDICAL ITEMS

### TYPHOID FEVER REDUCED IN RURAL COMMUNITIES

Reduction in typhoid fever and improvement in sanitary conditions have followed the intensive investigations of rural communities carried on by the United States Public Health Service in co-operation with local and State health officers, according to the annual report of the Surgeon of that Service. During the past fiscal year 16,369 rural homes in eight different states were visited and many of them re-visited. In each of these homes information was obtained as to the prevalence of disease and unsanitary conditions and a complete sanitary survey of the premises conducted. This was followed by reinspections to determine if remedial measures had been instituted. In but a relatively small percentage of the cases did the persons concerned, after having their attention drawn to the danger of a particular unhygienic condition, fail to inaugurate corrective measures. Stimulus was given to the work by means of public lectures, the formation of active sanitary organizations, and the enlisting of all public spirited citizens in the campaigns for reform. Public buildings were also inspected and local authorities given expert advice in solving such sanitary problems as the disposal of excreta, the prevention of soil pollution, and the maintenance of pure water supplies.

The surveys made during the year 1914 had shown that in rural communities less than one per cent of the homes had sanitary toilets and that more than fifty per cent. of the people were using water from polluted sources. This condition, according to the Public Health Service, made the rural sanitation question loom large among the matters vitally affecting the welfare of the nation. Following these studies and as a result of the interest aroused, the typhoid fever rate, an excellent indicator of the sanitary status of a community, has in some places frequently been cut to one quarter of its previous figure. In Berkeley County, West Virginia, the cases of typhoid fever were reduced from 249 to 40 in one year. In Orange County, North Carolina, the rural sanitation campaign resulted in a reduction of the cases from 59 to 17.

The tangible results of operations in rural sanitation indicate that marked advancement in maintaining hygienic and satisfactory surroundings in country districts is possible by the application of the common principles of preventive medicine. Unsanitary conditions exist largely because they are not known to be such. Actual demonstrations of their harmfulness, together with definite recommendations for their correction, remain one of the most gratifying and successful methods for instituting reforms and has been, in the experience of the Public Health Service, invariably accompanied by definite and measureable results.

---

### A SAFE AND RELIABLE CARDIAC TONIC

It is a matter of common observation in the treatment of functional cardiac disorders that all remedies must be avoided that tend to cause reactionary exhaustion or depression following their initial effect. To use a homely but more or less apt illustration, the driver of an unruly high strung horse should not pull it up suddenly or harshly, but always gently and firmly. Therefore, when the heart is irritable or unruly, as it were, it can best be regulated by a remedy that will accomplish this purpose in a gentle yet firm uniform manner. Clinical experience extending over many years has shown that Cactina is such a remedy and can be relied upon as a most satisfactory tonic in all functional cardiac derangements.

At any rate the more one uses Cactina Pillets in the heart cases constantly being met in every-day practice, the more one grows to realize the value of this remedy. Prompt and positive in effect, Cactina has the great advantage of being perfectly safe and free from any tendency to cumulative action. It should, however, be constantly borne in mind that Cactina is not a powerful cardiac stimulant, but on the contrary is a tonic that acts by sustaining and steadying the heart.

Cactina has no contra-indications, and the experience of many thousands of competent physicians has conclusively shown that it can be used under any and all conditions with implicit confidence not only in its freedom from harmful or unpleasant effects, but equally in its capacity to support and strengthen the heart's action and thus help it to do its work.

---

### CORYSA—ACUTE NASAL CATARRH.

This condition is manifested by a local congestion of the nasal mucous membrane, with an infiltration of serum into the tissues and later an exudation on the part of the mucous membrane.

The local treatment calls for a remedy capable of relieving the engorgement by exosmosis, which can never be achieved by the use of acid or astringent preparations.

The use of Glyco-Thymoline in these cases purges the mucous membrane, relieving the congestion, and then by stimulating the local capillary circulation to renewed activity prevents a re-engorgement.

